# TRPM3 Expression in Mouse Retina

**DOI:** 10.1371/journal.pone.0117615

**Published:** 2015-02-13

**Authors:** R. Lane Brown, Wei-Hong Xiong, James H. Peters, Merve Tekmen-Clark, Iwona Strycharska-Orczyk, Brian T. Reed, Catherine W. Morgans, Robert M. Duvoisin

**Affiliations:** 1 Department of Integrative Physiology and Neuroscience, Washington State University, Pullman, Washington, United States of America; 2 WWAMI Medical Education Program, Washington State University, Pullman, Washington, United States of America; 3 Department of Physiology & Pharmacology, Oregon Health & Science University, Portland, Oregon, United States of America; NIH/NEI, UNITED STATES

## Abstract

Transient receptor potential (TRP) channels constitute a large family of cation permeable ion channels that serve crucial functions in sensory systems by transducing environmental changes into cellular voltage and calcium signals. Within the retina, two closely related members of the melastatin TRP family, TRPM1 and TRPM3, are highly expressed. TRPM1 has been shown to be required for the depolarizing response to light of ON-bipolar cells, but the role of TRPM3 in the retina is unknown. Immunohistochemical staining of mouse retina with an antibody directed against the C-terminus of TRPM3 labeled the inner plexiform layer (IPL) and a subset of cells in the ganglion cell layer. Within the IPL, TRPM3 immunofluorescence was markedly stronger in the OFF sublamina than in the ON sublamina. Electroretinogram recordings showed that the scotopic and photopic a- and b-waves of TRPM3^-/-^ mice are normal indicating that TRPM3 does not play a major role in visual processing in the outer retina. TRPM3 activity was measured by calcium imaging and patch-clamp recording of immunopurified retinal ganglion cells. Application of the TRPM3 agonist, pregnenolone sulfate (PS), stimulated increases in intracellular calcium in ~40% of cells from wild type and TRPM1^‑/‑^ mice, and the PS-stimulated increases in calcium were blocked by co-application of mefenamic acid, a TRPM3 antagonist. No PS-stimulated changes in fluorescence were observed in ganglion cells from TRPM3^-/-^ mice. Similarly, PS-stimulated currents that could be blocked by mefenamic acid were recorded from wild type retinal ganglion cells but were absent in ganglion cells from TRPM3^-/-^ mice.

## Introduction

The retina is a small neural network which converts light stimuli into parallel pathways of neural activity that are propagated to the brain. The diversity of retinal neurons and their functional connectivity is steadily being uncovered, including the elucidation of the molecular organization of retinal synapses and the signal transduction pathways that modulate transmission. Key to the function of neuronal circuits in the retina and brain are ion channels that maintain resting membrane potential and carry neural signals by their controlled opening and closing.

Transient receptor potential (TRP) channels form a large family of cation permeable ion channels that play a role in many sensory systems [1]. The retina expresses TRP channels from each of the major families, including the classic TRPs (TRPCs), the vanilloid receptor TRPs (TRPVs), and the melastatin TRPs (TRPMs) [2]. TRPCs function as receptor-operated channels and are thought to activate calcium signaling pathways and modulate cellular excitability. In the retina, TRPC6 and TRPC7 mediate the melanopsin-activated depolarizing current in intrinsically photosensitive retinal ganglion cells [3], [4]. Channels of the TRPV family are best known as heat-activated channels, including TRPV1, the well-characterized capsaicin receptor involved in thermal nociception [5]. Channels of the TRPM family serve diverse functions; the best characterized member of this family, TRPM5 is known to be involved in taste sensation, and TRPM8 is activated by cool temperatures and is thought to be responsible for the “cool” sensation elicited by menthol [6]. TRPM3 was recently shown to be activated by moderate heat, as well as the steroid pregnenolone sulfate (PS) [7]. TRPM3 channels are found in islet cells of the pancreas, where they are though to regulate insulin secretion [7], and were recently discovered in temperature-sensitive neurons of the dorsal root ganglia [8]. TRPM3 expression has been reported in the brain, both in neurons and oligodendrocytes [9] as well as the peripheral nervous system, in trigeminal and dorsal root ganglia [10]. TRPM3 is also expressed in testes and in human, but not mouse, kidneys [11], [12].

Two of the TRPM channels, TRPM1 and TRPM3, are highly expressed in retina [2], [13], [14]. Of the eight known TRPM channels, TRPM1 and 3 are the two most closely related, sharing 57% sequence identity [12]. The proposed topology of TRPM channels is for a large intracellular amino (N)-terminal domain, 6 transmembrane segments, a re-entrant loop forming the pore of the channel, and a large, intracellular carboxy (C)-terminal region. Heterologous expression studies indicate that TRPM3 mediates an outwardly rectifying cation current [15]. TRPM3 currents are activated by moderate heat, augmented by extracellular Zn^2+^, and inhibited by intracellular Mg^2+^ [16]. Pharmacologically, TRPM3 channels are activated by the neurosteroid PS and inhibited by mefenamic acid (MA) [7], [17].

TRPM1 is exclusively expressed by ON-bipolar cells where it mediates the depolarizing light response of ON-bipolar cells via negative coupling to the retina-specific metabotropic glutamate receptor, mGluR6 [18], [19], [20]. It is also expressed by the ciliary body of the eye and by melanocytes in the skin [2], [21]. In the eye, TRPM3 is expressed in the iris, retinal pigment epithelium (RPE) and the neural retina, as determined by expression sequence tags (ESTs), *in situ* hybridization and immunohistochemistry [2], [22], [23], but their precise localization and function remain unknown. Here, we localize TRPM3 to inner retinal synapses and ganglion cells, and demonstrate functional expression of TRPM3 in a subset of retinal ganglion cells.

## Materials and Methods

### Animals

All animal procedures were in accordance with the National Institutes of Health guidelines and were approved by the Institutional Animal Care and Use Committees at Oregon Health & Science University and Washington State University. Heterozygote TRPM3^+/tm1Lex^ (MGI:3528953) mice, originally produced by Lexicon Pharmaceuticals (The Woodlands, TX), were obtained from the Mutant Mouse Regional Resource Center (University of North Carolina, Chapel Hill, NC). Homozygous TRPM3^-/-^ and TRPM3^+/+^ (wild type; WT) mice were produced by breeding mice generated from heterozygote parents. Genotyping was performed as recommended by Lexicon Pharmaceuticals. Animals aged from P21 to P50 were used in this study.

### Molecular cloning and expression in transfected Chinese Hamster Ovary cells

Total RNA was isolated from two dissected mouse retina using High Pure RNA Tissue Kit (Roche Applied Science, Indianapolis, IN) according to the manufacturer’s procedure. First strand cDNA was synthesized using SuperScript III First-Strand Synthesis System for RT-PCR (Invitrogen, Carlsbad, CA) and oligo-dT as primer. Sense and antisense primers were designed according to the mouse TRPM3 GenBank sequence NM_001035239, spaced 900–1700 bp apart with 100–200 bp overlaps for overlap extension. The primer sequences were (5’ to 3’): 1s, CAAGGGCCAGGCTGCTAAGAATG; 13s, AGGCCACCAGGACATTGATTTAGC; 14as, GGCCACTGCTGCCCGTAAATAAAG; 25s, TGGAACGTCACAGACCTCATCGC; 25as, TTCACACAGTAGATGACCCTCCCG; 30as, GATGTCCACGGTCTGGAGTGAAGC; 30bas, ACTCAAGGAAGGGGGAACGTGGTG. Polymerase chain reactions were performed using high fidelity *Pfu* polymerase (Stratagene-Agilent, Santa Clara, CA), reaction products were cloned using CloneJET PCR Cloning Kit (Fermentas, Glen Burnie, MD). Overlap extension PCR was used to generate a full length TRPM3 cDNA fragment, but attempts to subclone this construct were unsuccessful. Thus, silent *Sal*I and *Sac*I restriction sites were introduced by PCR, as well as terminal *Hin*dIII and *Age*I sites with the following primers: Sal-s, GAGTcGACATCGCTCGCAGCCAGA; Sal-as, ATGTCgACTCTGTTCCAGGCTAAAGC; Sac-s, TAGACCCcGCgGGTGAGGAGACCATATCCCCAAC; Sac-as, CTCACCcGCgGGGTCTATACTCTCTTGAAGTTTG; Hin, gAAGCTTaagaATGGGCAAGAAGTGG; Age, cgaccggtTTGTGCTTGCTTTCAAAGCTATGAAAGG. Restriction fragments were subcloned sequentially into pEGFP-N1 vector (Clontech, Mountain View, CA) to generate a full-length construct. The TRPM3-mCherry construct was prepared by replacing EGFP by mCherry. Nucleotide sequences were determined by the Vollum Institute DNA sequencing core at OHSU.

Chinese hamster ovary (CHO) cells were cultured in DMEM media supplemented with 10% fetal bovine serum and a penicillin/streptomycin mixture at 37°C in a humidified incubator at 5% CO_2_. Cells were transfected with a plasmid encoding a full-length mouse retina TRPM3-mCherry fusion construct under control of the CMV promoter using Lipofectamine (Invitrogen), according to the manufacturer’s recommendations. For Ca^2+^ imaging, CHO cells were cultured and transfected on coverslips coated with poly-L-lysine and imaged 1–3 days post-transfection. For whole cell electrophysiological recordings, cells were mechanically detached from the culture plate 2–5 days following transfection, triturated to obtain a single-cell suspension, dropped into an open recording chamber, and allowed to settle to the bottom.

### Immunohistochemistry

To produce a rabbit antiserum against TRPM3 a 390 bp-long *Bam*HI-*Xho*I restriction fragment was generated by PCR using the following primers: Bam, ccggatccTACTACGCCAACTTTGGG; Xho, gactcgagtcaAAGCTTGTCACTGATGGAG and subcloned into pET28a (Novagen, EMD Millipore, Billerica, MA). The 17,751 kD His-tagged fusion protein was purified by preparative SDS-PAGE and used to immunize 2 rabbits (Covance, Princeton, NJ).

Retina section were prepared and immunohistochemistry was performed as described previously [24], [19]. The optimal conditions were established as a 1:16,000 dilution of the antiserum. Double labeling used mouse monoclonal antibodies against calretinin (Chemicon-Millipore, Temecula, CA) and Brn3a (EMD Millipore). Secondary antibodies coupled to Alexa-488 or Alexa-594 (Invitrogen, Carlsbad, CA) were used at 1:2000 dilutions. Specificity of the anti-TRPM3 antiserum was verified by immunostaining of retinal sections from TRPM3^-/-^ mice. Images were acquired with an Olympus FluoView FV1000 confocal microscope using a 60X/1.42 oil immersion objective. Images were adjusted for brightness and contrast using Pixelmator (London, UK).

### Electroretinogram recording

Electroretinograms (ERGs) were recorded from 5 WT and 4 TRPM3^-/-^ mice. Mice were dark-adapted overnight (>12 hrs) and prepared for recording under dim red light. Mice were anesthetized with an intraperitoneal injection of ketamine and xylazine (100:10 mg/kg) and maintained with supplemental 30:3 mg/kg anesthesia injections approximately every 35 minutes. Body temperature was maintained at 36–37°C by placing the mouse on a circulating-water heating pad. Before ERG recording, the pupils were dilated with 2.5% phenylephrine and 1% tropicamide and the cornea anesthetized with 1.0% proparacaine. A custom made cone placed over the snout allowed delivery of O_2_ which helped minimize breathing artifacts during recording. The ERG was recorded from a custom-made contact lens electrode placed against the cornea with a small drop of 1% methylcellulose. A platinum needle electrode bent at 90°, placed in contact with the center of the cornea with a small amount of 2.5% methylcellulose gel, served as the active electrode. Similar platinum reference and ground electrodes were placed in the forehead and tail, respectively. Mouse and heating plate were then advanced into a Ganzfeld diffusing sphere and light stimuli were provided by custom made LED photoflash units. The flash intensity could be controlled by altering flash duration (between 30 μsec and 1 msec) and current through the LED. A 3.0 log unit neutral density filter was used to further extend the flash intensity range. Flash intensities were measured using a photometer (Model IL1700; International Light, Newburyport, MA) fitted with a scotopic filter in integrating mode that gave results as scotopic (sc) candela second per square meter (cd-s/m^2^). Scotopic and photopic ERGs were amplified at a gain of 5000, and band-pass filtered (0.1 to 1k Hz). Data were acquired with a data acquisition board (sampling rate: 10 kHz; National Instruments, Austin, TX). Traces were recorded with customized software (ERGTool, Dr Richard Weleber, Casey Eye Institute, Portland, OR).

### Immunopurification of mouse retinal ganglion cells

Retinal ganglion cells were dissociated and immunopurified using a procedure modified from those previously described by Hartwick, et al. [25] and Barres et al. [26]. Mice (C57BL/6, TRPM1^-/-^, and TRPM3^-/-^) were fed ad libitum and housed under a 12 hr light/dark cycle. In brief, four to eight mice of the desired genotype, aged 21–60 days, were anesthetized with isoflurane (Butler Schein, Isothesia, North Dublin, OH) and euthanized by cervical dislocation. Eyes were enucleated, the anterior segment and vitreous were removed and discarded. During the process of dissection, isolated retinas were initially stored in Hibernate-A medium (Springfield, IL, USA), supplemented with 2% B27 mixture (Invitrogen) and 10 μg/ml gentamicin, before being transferred to a dissociation solution containing 165 units of papain (Worthington Biochemicals, Lakewood, NJ), 1 mM L-cysteine, and 0.004% DNase in 10 ml of Ca^2+^/Mg^2+^-free Dulbecco’s phosphate-buffered saline (DPBS, Invitrogen). After a 30 min. incubation at 37°C, the retinas were allowed to settle and the dissociation solution was removed and replaced with 10 ml of an ovomucoid inhibitor solution (LowOvo), containing 1.5 mg/ml ovomucoid (Roche), 1.5 mg/ml bovine serum albumin (BSA), and 0.0004% DNase in DPBS containing Ca^2+^ and Mg^2+^. Following gentle mechanical trituration with a P1000 Pipetman (Gilson) to disperse the cells, the retinal suspension was collected via centrifugation (200 x g for 10 min. at RT), and washed in “HighOvo” inhibitor solution, composed of DPBS containing 10 mg/ml ovomucoid and BSA. Dissociated retinal cells were once again collected by centrifugation, and resuspended in DPBS containing 0.2 mg/ml BSA and 5 μg/ml insulin in preparation for immunopanning. To remove large cell clumps, the retina cell suspensions were passed through a 40 μm mesh cell strainer (BD Falcon). Macrophages, endothelial cells, and other contaminating blood cells were removed by incubating the retinal suspension for 0.5 hr on a 100 mm Petri dish that had been coated with *Griffonia simplicifolia* lectin I (Vector Laboratories, Burlingame, CA) Next, the cell suspension was incubated on a 100 mm Petri dish that had been sequentially coated with goat anti-mouse IgM (Jackson ImmunoResearch, West Grove, PA) and mouse anti-mouse Thy-1.2 (Serotec MCA02R; Note: Use of anti-Thy1.2 instead of anti-Thy1.1 is the primary modification required for the successful immunopurification of mouse retinal ganglion cells compared to rat retinal ganglion cells [27]). Following a 2 hr. incubation, the plate was washed six times with DPBS to remove non-adherent cells, and purified retinal ganglion cells were released with 0.125% trypsin in DPBS, which was subsequently quenched by the addition of 10 ml of 30% FBS in Neurobasal A.

Purified retinal ganglion cells were collected by centrifugation, and then plated onto square Poly-D-lysine-coated coverslips or onto round poly-D-lysine/laminin coated coverslips (12 mm; BD BioSciences, Bedford, MA, USA), and cultured in cultured in Neurobasal-A media, containing 2% B27 Supplements, 1 mM glutamine, 50 ng/ml brain-derived neurotrophic factor (BDNF), 10 ng/ml ciliary neurotrophic factor, 5 μM forskolin, and 10 μg/ml gentamicin. Cultures were maintained at 37°C in a humidified tissue culture incubator in 5% CO_2_. Electrophysiological and Ca^2+^-imaging experiments were performed following 1–6 days of culture in this medium.

### Calcium imaging

Calcium measurements were made using the fluorescent Ca^2+^ indicator Fura-2. TRPM3-transfected CHO cells, or immunopurified retinal ganglion cells on coverslips, were loaded with 1 μM Fura-2-AM for one hour at room temperature followed by a 15 min wash to allow for de-esterification. For figures, coverslips were used to form the bottom plate of a closed 1 ml-volume chamber (Warner Instruments), mounted onto the stage of an inverted Nikon Eclipse TE2000–2 microscope, and constantly perfused by a gravity-driven system at 5 ml min^-1^. Experiments were performed at room temperature (21°C) in a physiological saline bath (in mM: 140 NaCl; 5 KCl; 2 CaCl_2_; 1 MgCl_2_; 6 glucose; 10 HEPES with pH adjusted to 7.4 with NaOH). High K^+^ bath solution (HiK) contained 55 mM KCl with an equimolar reduction of NaCl. Cells containing Fura-2 were alternately excited with 340 and 380 nm light using a Sutter Lamda 10B with SMART Shutter and a Siskiyou Instruments filter wheel (Grants Pass, OR) and fluorescence monitored at 510 nm. Data points were collected at 6 sec time intervals. Ratios of fluorescence intensity were converted to calcium concentrations on-line using logarithmic fit parameters to a standard curve generated by Ca^2+^-EGTA titration. Data collection was controlled with MetaFluor software. Additional cells were plated on 12-mm round coverslips and imaged in an open chamber on an upright Olympus BX-51W microscope. Illumination was provided by a Sutter DG-4 (Sutter Instrument Company, Novato, CA), and data acquisition was controlled by Elements imaging software (Nikon Instruments). Results were similar for both imaging systems; we found, however, that background fluorescence and perfusion rates were more variable in the open chamber.

### Electrophysiological recordings

Recording solutions for TRPM3-transfected CHO cells were similar to those used by Lambert et al. [28]. The pipet solution contained (in mM): 80–90 CsAsp, 45 CsCl, 4 Na_2_ATP, 10 BAPTA, 5 EDTA, and 10 HEPES. The pH was adjusted to 7.2 with CsOH (adding ∼60 mM Cs^+^ to the solution), and the osmolality was adjusted to values within the range of 305–320 mOsm; the bath solution contained (in mM): 145 NaCl, 3 KCl, 10 CsCl, 2 MgCl_2_, 2 CaCl_2_, and 10 HEPES, pH 7.2. For recordings from cultured retinal ganglion cells, the pipet solution contained (in mM): 110 Cs-gluconate, 10 NaCl, 5 Na-HEPES, 1 Cs-EGTA, 1 Na-ATP, 0.1 Na-GTP, and 10 QX-314. Cesium was used in place of potassium to block voltage-gated potassium currents and thereby improve the quality of the voltage clamp at positive potentials. QX-314 was included to block voltage-dependent sodium channels and abolished all spiking activity within a few minutes of establishing the whole-cell configuration. To better match the HBSS-based solution used for Ca^2+^-imaging, the bath solution for whole-cell recording contained (in mM): 137 NaCl, 5.4 KCl, 0.25 Na_2_HPO_4_, 0.44 KH_2_PO_4_, 1.3 CaCl_2_, 1.0 MgSO_4_, 4.2 NaHCO_3_, 15 HEPES and adjusted to a pH of 7.4. Osmolarity was adjusted by dilution or by the addition of glucose. Cells were visualized on an inverted Nikon Diaphot microscope. Transfected CHO cells were identified by red fluorescence, whereas immunopurified retinal ganglion cells were randomly selected. Electrodes were pulled from 1.2 mm O.D. borosilicate glass using a Sutter P-97 puller, and had resistances from 3–5 mOhm. After obtaining the whole-cell configuration, CHO cells were lifted off the bottom of the chamber and placed before a three-port perfusion tube (Quick Step, Warner Instruments, Hamden, CT) to enable rapid solution exchange; cultured retinal ganglion cells remained on the coverslip following culture, and the perfusion tube was lowered to within 0.5 mm. Voltage-clamp recordings were performed at a holding potential of-60 mV with a Axon Instruments 700A patch-clamp amplifier (Molecular Devices, Sunnyvale, CA) connected to an Axon Instruments Digidata 1321A 16 bit A-D converter and a Dell Windows PC. Voltage ramps from-100 to +100 mV were applied every 20 s for a duration of 250 ms to examine current-voltage relations. Currents were digitally sampled at 10 kHz and low-pass filtered offline at 0.2–2 kHz with an 8-pole Bessel software filter. Data were analyzed offline with pClamp (Molecular Devices), and figures were prepared with SigmaPlot.

## Results

### TRPM3 isoforms expressed in the retina

**Fig 1:**
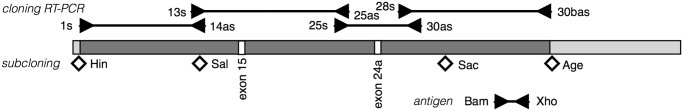
Isolation of a full-length TRPM3 cDNA from mouse retina by RT-PCR. Four segments of TRPM3 were amplified from retina cDNA. The primer’s exon and orientation (sense vs antisense) are indicated. The 5’ and 3’ untranslated regions are shown in light grey, the coding region in dark, and splice variation (see text) in white. Restriction enzyme sites used for sequential cloning of overlap extension products are indicated by diamonds. The location of the *Bam*HI-*Xho*I cDNA fragment encoding the antigen used to raise an anti-TRPM3 antiserum is shown.

Complementary DNA fragments encoding TRPM3 were obtained from mouse retina by RT-PCR, and a full-length TRPM3 cDNA was constructed by overlap extension (Fig. 1A). Among the seven splice variants previously described [15], [29], we found that retinal TRPM3 varied in its inclusion of the 36 bp-long exon 15. We also detected exon 24a in the mouse retinal TRPM3 cDNA, which differs from exon 24 in the splice donor site. The use of this shorter variant of exon 24 is interesting because the encoded amino acids are located in a region that is proposed to form the pore loop domain of TRP channels and has been shown to affect divalent cation selectivity of the channel {Oberwinkler et al 2005}.

### Expression of recombinant mouse TRPM3

**Fig 2:**
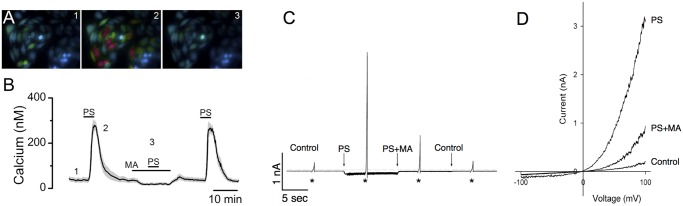
Expression of recombinant mouse retinal TRPM3. **A, B**) Ca^2+^ signals were recorded from CHO-K1 cells transiently transfected with mouse TRPM3 by Fura-2-based ratiometric imaging (n = 8 PS-responsive CHO cells). Signal from control solution (1) and Pregnenolone Sulfate (PS)-activated rises in calcium (2). The PS signal was abolished by pretreatment with mefenamic acid (MA; 3). In (**B**), black line is average, grey is SEM. C, D) CHO-K1 cells were transiently transfected with mouse mCherry-tagged TRPM3. PS activates an outwardly rectifying current in mCherry—TRPM3 transfected cells identified with mCherry fluorescence. PS-stimulated currents were measured from 11 cells; inward currents at -100 mV were 83 ± 13 pA (SEM) and outward currents were 2175 ± 128 pA at +100 mV. Non-transfected CHO cells show no measurable response to application of PS. The currents were blocked by MA. * in (**C**) indicate time when the holding potential was ramped from -100 to +100 mV to determine I–V relationship in (**D**).

CHO cells were transiently transfected with a plasmid encoding mouse TRPM3 fused at its C-terminus to the fluorescent protein, mCherry, to aid in the identification of transfected cells. Cells were loaded with the calcium indicator, Fura-2, and Ca^2+^ signals recorded by ratiometric imaging in response to perfusion with 50 μM Pregnelone Sulfate (PS), a TRPM3 agonist (Fig. 2A). The PS-driven Ca^2+^ signals were blocked with mefenamic acid (MA), a TRPM3 channel blocker [17]. Average Ca^2+^ signals and SEM are shown in Fig. 2B.

CHO cells were transiently transfected with mCherry-tagged mouse TRPM3 and currents measured by whole cell patch clamp recording. Application of PS activated an inward current in transfected cells but not in untransfected cells (Fig. 2C, D). The PS sensitive current was abolished in the presence of MA. The PS-activated current displayed outward rectification as previously shown for recombinant TRPM3 by Lambert et al. [28].

### Localization of TRPM3 in the retina

**Fig 3:**
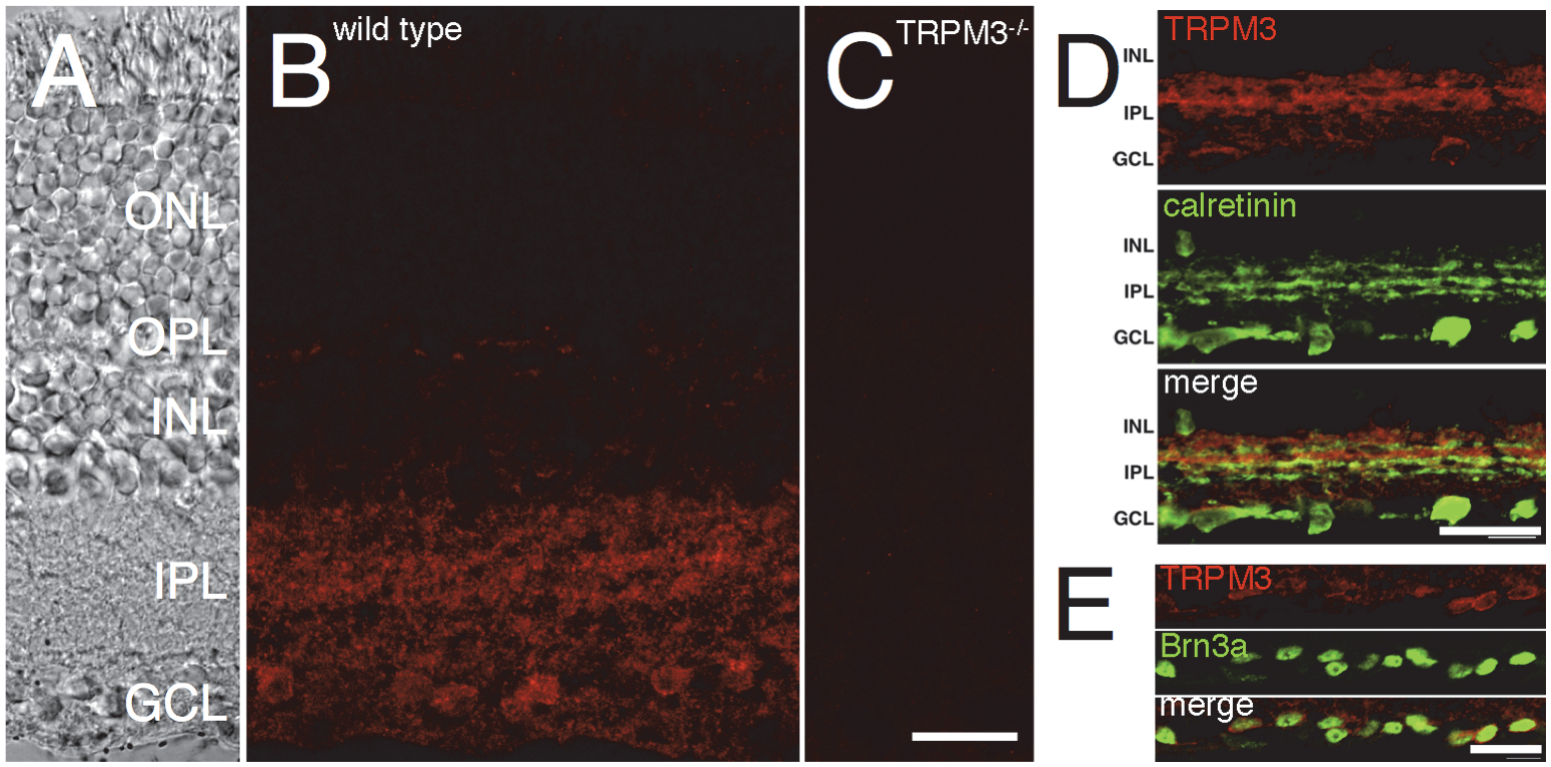
TRPM3 is expressed in the IPL and GCL of the mouse retina. Mouse retina sections were labeled by immunofluorescence for TRPM3. **A**) Transmitted light DIC image showing the retinal cell layers. **B**) TRPM3 immunoreactivity is observed in the IPL and GCL of a WT mouse retina. **C**) No immunoreactivity is detected in a retinal section from a TRPM3^-/-^ mouse. **D**) Transverse retinal sections of the inner retina double-labeled for TRPM3 (top, red) and calretinin (middle, green). Visible in the merged image (bottom), the outer, OFF half of the IPL (sublamina a) is more strongly labeled than the inner, ON, sublamina b. **E**) Imaging of the ganglion cell layer, double-labeled for TRPM3 (top, red) and Brn3a (middle, green). The merged image (bottom) shows that Brn3a-positive ganglion cells express TRPM3. Abbreviations are as follows: ONL, outer nuclear layer; OPL, outer plexiform layer; INL, inner nuclear layer; IPL, inner plexiform layer; GCL, ganglion cell layer. Scale bars = 20 μm.

For immunofluorescent localization of the TRPM3 channel in the retina, antiserum was generated against a peptide corresponding to C-terminus of mouse TRPM3. Reactivity of the antiserum with TRPM3 was confirmed by western blotting and immunofluorescent labeling of HEK293 cells transfected with a plasmid encoding a GFP-TRPM3 fusion protein (not shown). TRPM3 antiserum was applied to retina sections from WT and TRPM3^-/-^ mice. In the WT retina, immunofluorescence was strong over the inner plexiform and ganglion cell layers (Fig. 3B). Weak immunofluorescence was observed in the inner nuclear layer and outer plexiform layer. No immunoreactivity was observed in the TRPM3^-/-^ retina (Fig. 3C). Within the inner plexiform layer (IPL), TRPM3 immunoreactivity was stronger in the OFF sublamina compared to the ON sublamina. This is apparent in double labeling for TRPM3 and calretinin, which labels three bands in the IPL (Fig. 3D) [30]. Within the ganglion cell layer, a subset of cells (~40%) were intensely labeled. From the large size of the cell bodies and their double labeling with Brn3a these are most likely ganglion cells (Fig. 3E). There are however a few TRPM3-positive cells that are not labeled by Brn3a. These could be displaced amacrine cells, or Brn3a-negative ganglion cells.

### Effects of TRPM3 on the electroretinogram

**Fig 4:**
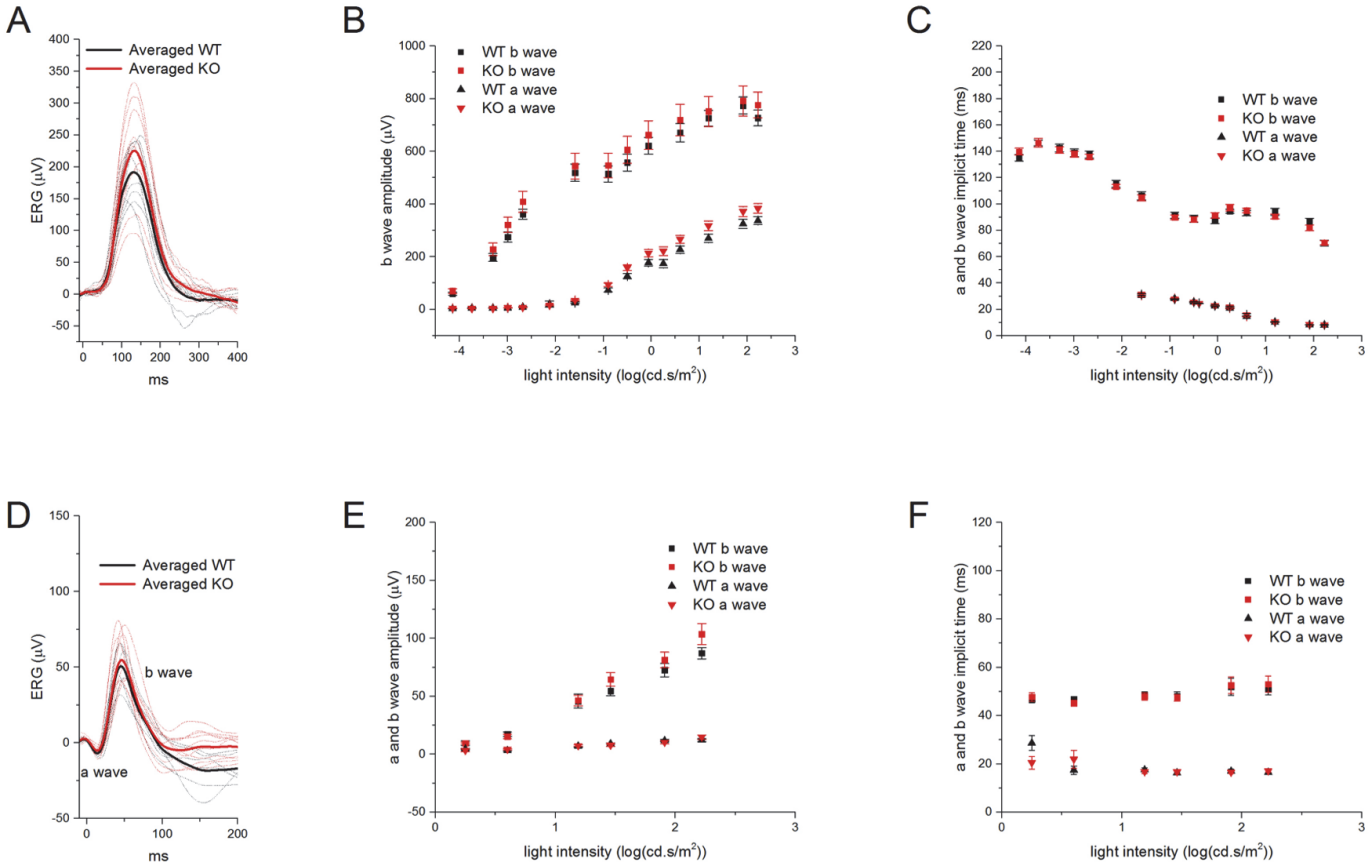
Comparison of the ERGs of TRPM3^-/-^ and TRPM3^+/+^ mice. **A**) Traces of low pass-filtered scotopic ERGs from TRPM3^+/+^ (black) and TRPM3^-/-^ (red) mice. The flash intensity was-3.3 log(cd-s/m^2^). Each dashed line is the averaged response from one eye. The thick lines are averages for each genotype. The amplitude (**B**) and implicit time (**C**) of the scotopic a- (▲) and b- (■) waves at different light intensities are plotted for TRPM3^+/+^ (black) and TRPM3^-/-^ (red) mice. Data points show the mean with the standard error of the mean. **D**) Traces of low pass-filtered photopic ERGs from TRPM3^+/+^ (black) and TRPM3^-/-^ (red) mice. The flash intensity was 1.91 log (cd-s/m^2^) under 100 cd/m^2^ white light background. Each dashed line is the averaged response from one eye. The thick lines are averages for each genotype. The amplitude (**E**) and implicit time (**F**) of the photopic a- (▲) and b- (■) waves at different light intensities are plotted for TRPM3^+/+^ (black) and TRPM3^-/-^ (red) mice. Data points show the mean with the standard error of the mean.

Comparison of ERG recordings from TRPM3^+/+^ and TRPM3^-/-^ litter mates did not reveal major differences in either scotopic (Fig. 4A-C) or photopic flash responses (Fig. 4D-F). Photopic b-wave amplitudes and implicit times were the same in the TRPM3^-/-^ and WT, arguing against a role for TRPM3 in generating the ON-bipolar cell light response.

### Pregnenolone sulfate activates TRPM3-dependent calcium signaling in mouse retinal ganglion cells

**Fig 5:**
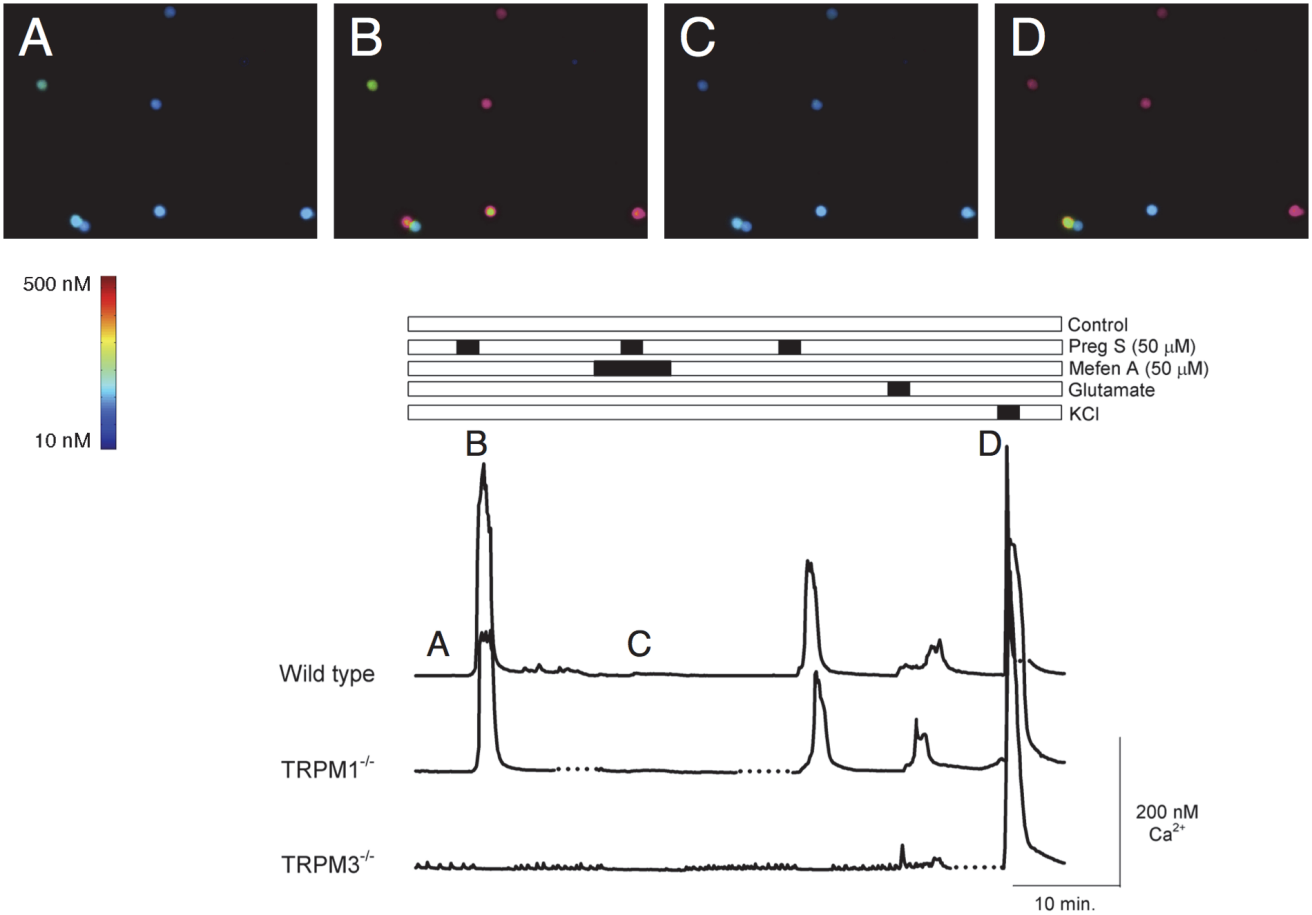
TRPM3-dependent intracellular calcium increases in mouse retinal ganglion cells. Ca^2+^ signals were recorded from immunopanned ganglion cells by Fura-2-based ratiometric imaging. Dotted lines indicate where gaps were introduced in some traces to facilitate proper alignment. Greater than 50% of ganglion cells from WT mice responded to Pregnenolone Sulfate (PS)-activated rises in calcium (**B**) compared to baseline (**A**); the PS signal was abolished by pretreatment with mefenamic acid (MA; **C**). In the TRPM3^-/-^ mice, no cells produced a large response to PS; a few cells, however, gave a small response that was not blocked by MA. At the end of the experiment, cells were depolarized with KCl perfusion to verify viability (**D**).

Retinal ganglion cells were purified from the retina of 3–6 week old mice by immunopanning with anti-Thy1.2 antibodies. Ca^2+^ signals were recorded by Fura-2-based ratiometric imaging (Fig. 5). Greater than 50% of ganglion cells from WT mouse retina (94 of 167) responded to bath application of 50 μM PS with an increase in intracellular Ca^2+^ larger than 100 nM (417 nM ± 63 nM (SEM)). Similar results were obtained with retinal ganglion cells purified from retinas of TRPM1^-/-^ mice (18 of 32 cells; 389 ± 27 nM). The PS signal was abolished by pretreatment with 50 μM MA (89 of 94 PS-responsive cells in the WT retina). In cultures prepared from TRPM3^-/-^ mice, no cells produced a response to PS larger than 50 nM (0 of 37); a few cells, however, gave a small response that was not blocked by MA. In order to confirm that the non-responsive cells were viable, they were challenged by the application of 1 mM glutamate followed by depolarization with a high K^+^ solution; in the majority of cells, both PS-responsive and non-responsive, bath application of either glutamate or a high K^+^ solution elicited an elevation in intracellular Ca^2+^, demonstrating that the majority of cells in our preparation, including those that did not respond to PS, were viable.

### Pregnenolone sulfate activates TRPM3 currents in mouse retinal ganglion cells

**Fig 6:**
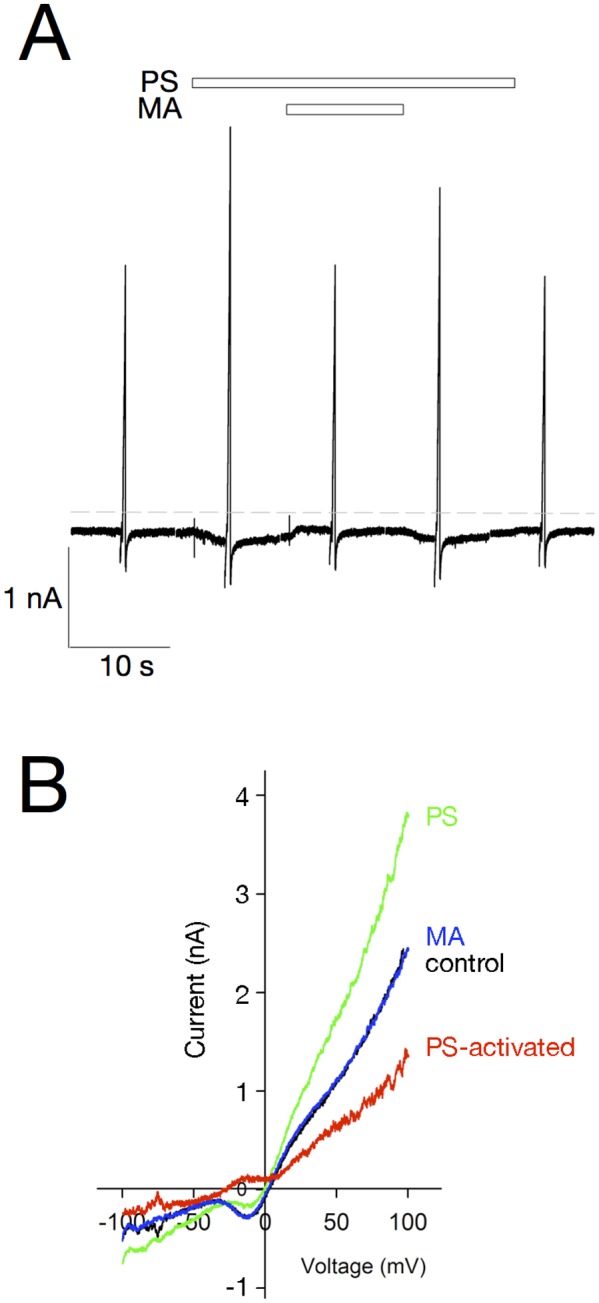
Pregnenolone sulfate activates a TRPM3-dependent outwardly rectifying current in isolated ganglion cells. Whole cell currents were recorded from isolated retinal ganglion cells. Holding potential was-60 mV; near vertical current ramps were generated by a voltage ramp from-100 to +100 mV over 250 ms. **A**) Time course illustrates activation of current by focal extracellular application of PS, and the subsequent inhibition by co-application of MA. **B**) Current-voltage relation of currents from the cell shown in (**A**). The PS-activated current is PS-Control and displays strong outward rectification characteristic of TRPM3.

Whole cell currents of dissociated, immunopurified retinal ganglion cells were recorded in response to voltage ramps from-100 to +100 mV over 250 ms (Fig. 6A). PS-activated currents were obtained by subtracting currents recorded in control solution from those recorded in the presence of 50 μM PS. These PS-activated currents were blocked by 50 μM MA and display strong outward rectification characteristic of TRPM3 (Fig. 6B). In dissociated, immunopanned retinal ganglion cells, PS elicited a measurable current in 7 of 20 cells, and this PS-activated current was inhibited by MA in 6 of 7 PS-responsive cells. The rectification and reversal potential of the PS-activated currents were somewhat variable, likely due to non-selective effects of PS on other retinal ganglion cell ion channels. In the recording shown in Fig. 6B, the PS-activated current appears to reverse at about-20 mV. This positive “bulge” in the current-voltage relation may result from the inhibition of a PS-sensitive Ca^2+^ current as previously described [31].

## Discussion

TRPM1 and TRPM3 are closely related ion channels that can function as heteromultimers [28], and both are highly expressed in retina [2]. TRPM1 has been shown to have a critical function in generating the light response of ON-bipolar cells [18], [19], [20], yet little is known about TRPM3 in the retina. Thus, we sought to determine the distribution and function of TRPM3 in the mouse retina.

At least seven TRPM3 splice variants have been discovered, which vary in their ion selectivity and other biophysical properties [29]. In mouse retina, we identified cDNA fragments encoding the TRPM3α2 and TRPM3α3 isoforms (nomenclature according to [15]), corresponding to the human TRPM3a and TRPM3b variants [12]. Both TRPM3α2 and TRPM3α3 differ from TRPM3α1 (human TRPM3c) by their use of an alternate exon 24 donor splicing site (exon 24a), which results in a 36 bp shorter mRNA, and a 12 amino acid deletion. This difference is expected to lie in the region lining the ion conducting pore and affect the ion selectivity of the channels, with TRPM3α2 having higher Ca^2+^ and Mg^2+^ permeability than TRPM3α1 [15]. This feature remains to be investigated in the retinal clone.

A full-length cDNA encoding TRPM3α2 from mouse retina was expressed as a fusion with mCherry in transfected CHO cells and its functional integrity confirmed by calcium imaging and whole cell patch-clamp recordings. Transfected cells expressing TRPM3-mCherry generated large Ca^2+^ transients when exposed to bath-applied PS that were blocked by MA. Furthermore, expression of TRPM3-mCherry resulted in PS-activated cationic currents that displayed strong outward rectification and were inhibited by co-application of MA similar to previously reported results [28].

Our immunohistochemical studies showed that TRPM3 is expressed in the IPL, where bipolar cell axon terminals, amacrine cells processes and ganglion cell dendrites form synapses, and in the GCL, where displaced amacrine and ganglion cell bodies are localized. In the GCL, TRPM3 is expressed in Brn3a-positive ganglion cells, but also in a few Brn3a-negative cells. These Brn3a-negative cells could be displaced amacrine cells or intrinsically photosensitive retinal ganglion cells (ipRGCs) involved in the photoentrainment of circadian rhythms [32]. In the IPL, TRPM3 labeling appears more intense in the outer half (sublamina a) where OFF synapses occur, compared to the inner half (sublamina b), where ON synapses, including rod bipolar cell synapses are located. TRPM3 immunofluorescence revealed a subset of ganglion cell bodies, ~40%, that were labeled with greater intensity, possibly corresponding to ganglion cells whose dendrites stratify in sublamina a of the IPL (i.e. OFF ganglion cells).

This distribution of TRPM3 immunofluorescence is consistent with our electrophysiological findings. ERG recordings indicate that TRPM3 has a negligible effect on either scotopic or photopic a-waves and b-waves, consistent with the absence of TRPM3 immunostaining in the photoreceptors, responsible for the generation of the a-wave, and weak immunostaining in the outer plexiform layer, where much of the b-wave is generated. Expression of TRPM3 by ganglion cells is confirmed by our measurements of PS-stimulated increases in intracellular Ca^2+^ in dissociated retinal ganglion cells. That this modulation of intracellular Ca^2+^ is mediated by TRPM3 is confirmed by the absence of such modulation in ganglion cells from TRPM3-deficient mice. It is notable that about 50% of dissociated ganglion cells responded to PS. One possibility is that these PS-responsive cells represent the ganglion cells with greater TRPM3 immunostaining and dendrites stratifying in the outer half of the IPL, although it is also possible that some of the TRPM3 immunostaining in the IPL is on amacrine cell processes.

Patch-clamp electrophysiological recordings of ganglion cells demonstrated the presence of an outwardly rectifying current, regulated by PS and blocked by the antagonist MA, typical of TRPM3 channels, and similar to the currents recorded from heterologously expressed TRPM3-mCherry. The inward current density at-60 mV was quite small in the ganglion cells compared to transfected cells, and was a less sensitive method, compared to Ca^2+^-imaging, of determining the percentage of retinal ganglion cells that express TRPM3.

The physiological function of TRPM3 in visual processing in the retina remains unclear, but our immunohistochemical data suggest a regulation of the OFF pathway and our electrophysiological and calcium imaging data suggest a direct effect on some ganglion cells. The neurosteroid PS, which is derived from cholesterol, and is a precursor of other steroids, such as glucocorticoids, androgens and estrogens, has many physiological effects, including neuroprotection, improved synaptic function, and improved cognitive function [33]. It is therefore possible that visual function is also modulated by neurosteroids, via TRPM3 stimulation. Recently, TRPM3 channels have been reported to have a second ion permeation pathway, sensitive to clotrimazole [34]. Whether an endogenous ligand can stimulate this pathway and whether retinal ganglion cell light responses would be modulated by clotrimazole or such an endogenous ligand remains to be studied.
